# Functional characterisation of *Schistosoma japonicum* acetylcholinesterase

**DOI:** 10.1186/s13071-016-1615-1

**Published:** 2016-06-10

**Authors:** Hong You, Geoffrey N. Gobert, Xiaofeng Du, Gabor Pali, Pengfei Cai, Malcolm K. Jones, Donald P. McManus

**Affiliations:** Molecular Parasitology Laboratory, Infectious Diseases Division, QIMR Berghofer Medical Research Institute, Brisbane, Queensland Australia; School of Biological Sciences, Queen’s University Belfast, Belfast, UK; School of Veterinary Sciences, The University of Queensland, Brisbane, Queensland Australia

**Keywords:** *Schistosoma japonicum*, Acetylcholinesterase, Drug or vaccine target

## Abstract

**Background:**

Acetylcholinesterase (AChE) is an important metabolic enzyme of schistosomes present in the musculature and on the surface of the blood stage where it has been implicated in the modulation of glucose scavenging from mammalian host blood. As both a target for the antischistosomal drug metrifonate and as a potential vaccine candidate, AChE has been characterised in the schistosome species *Schistosoma mansoni*, *S. haematobium* and *S. bovis*, but not in *S. japonicum*. Recently, using a schistosome protein microarray, a predicted *S. japonicum* acetylcholinesterase precursor was significantly targeted by protective IgG1 immune responses in *S. haematobium-*exposed individuals that had acquired drug-induced resistance to schistosomiasis after praziquantel treatment.

**Results:**

We report the full-length cDNA sequence and describe phylogenetic and molecular structural analysis to facilitate understanding of the biological function of AChE (*Sj*AChE) in *S. japonicum*. The protein has high sequence identity (88 %) with the AChEs in *S. mansoni*, *S. haematobium* and *S. bovis* and has 25 % sequence similarity with human AChE, suggestive of a highly specialised role for the enzyme in both parasite and host. We immunolocalized *Sj*AChE and demonstrated its presence on the surface of adult worms and schistosomula, as well as its lower expression in parenchymal regions. The relatively abundance of AChE activity (90 %) present on the surface of adult *S. japonicum* when compared with that reported in other schistosomes suggests *Sj*AChE may be a more effective drug or immunological target against this species*.* We also demonstrate that the classical inhibitor of AChE, BW285c51, inhibited AChE activity in tegumental extracts of paired worms, single males and single females by 59, 22 and 50 %, respectively, after 24 h incubation with 200 μM BW284c51.

**Conclusions:**

These results build on previous studies in other schistosome species indicating major differences in the enzyme between parasite and mammalian host, and provide further support for the design of an anti-schistosome intervention targeting AChE.

## Background

Schistosomiasis remains one of the most insidious and serious of the tropical parasitic diseases of clinical and public health significance. Currently, there is no effective vaccine to prevent schistosomiasis [1] and treatment is dependent on praziquantel chemotherapy. Previous reports showed that human schistosomiasis could be treated using the drug metrifonate [2], which can disrupt the cholinergic system and neuromuscular signalling by targeting acetylcholinesterase (AChE). Metrifonate was, however, withdrawn from the market because of unacceptable toxicity to the host and variable efficacy against different schistosome species [3].

During the blood dwelling stages of schistosomes, acetylcholinesterase (AChE) is present on the parasite tegument membrane [4] and in the musculature [5], both in adults and schistosomula. A previous study implicated schistosome AChE in regulating glucose scavenging from the host [6]. It has been shown that the basal rate of glucose uptake in adult *Schistosoma haematobium* and *S. bovis* is about twice that in *S. mansoni* [7]. Indicative of the higher metabolic requirements for glucose in *S. haematobium* and *S. bovis*, relatively higher amounts of AChE activity are present on their teguments compared with *S. mansoni* [2]. These higher levels of AChE activity result in the recorded higher susceptibility to metrifonate [8]. It has also been shown that *S. mansoni* AChE antibodies can lead to efficient complement-mediated killing of schistosomula in vitro [9]. Importantly, the absence of cross-reactivity with human AChE further supports schistosome AChE as a suitable target for immunological attack [9].

AChE has been characterised from *S. mansoni*, S*. haematobium* and *S. bovis* [10, 11], but not in *S. japonicum*. Recently, using a schistosome protein microarray, a predicted *S. japonicum* acetylcholinesterase precursor (AY810792) was significantly targeted by protective IgG1 immune responses in *S. haematobium-*exposed individuals that had acquired drug-induced resistance to schistosomiasis after praziquantel treatment [12]. This observation further supports consideration of *S. japonicum* AChE (*Sj*AChE) as a suitable vaccine candidate against schistosomiasis.

The interaction between acetylcholine (ACh) and its receptor, the nicotinic acetylcholine receptor (nAChR), results in the opening of the ion channel in mammalian cells [7]. Schistosome AChE plays an important role in limiting this interaction as the inhibition of AChE mimics ligand excess and causes receptor desensitisation [11]. It has been shown that circulating concentrations of ACh can result in an increase in glucose uptake in schistosomes in vitro, and this effect is ablated in the presence of anti-acetylcholinesterase antibodies [7]. Furthermore, the influence of acetylcholine on glucose uptake in these worms can be modulated through inhibition of either tegumental AChE or nAChR [11]. nAChRs are ligand-gated ion channels within the nervous system that mediate the excitatory responses to acetylcholine. Three types of acetylcholine receptors have been identified in *S. haematobium:* ShAR1α (AY392150) [13, 14], ShAR1β (AY392151) [14] and ShAR2β [15]. It has been demonstrated that ShAR1α is located on the parasite surface and may contribute to the potentiation of the uptake of glucose from the host blood in response to circulating concentrations of ACh.

As the first step in determining the functional characteristics of AChE from *S. japonicum*, we present the isolated full-length sequence of the protein from this schistosome species, describe the distribution of the enzyme in schistosomula and adult worms, and show that the classic inhibitor of BW284c51 effectively suppresses AChE activity in adult worms in vitro.

## Methods

### Parasites

*Schistosoma japonicum* adult worms were collected by perfusion of female ARC Swiss mice infected percutaneously with 60 cercariae of *S. japonicum* (Anhui population, mainland China) shed from *Oncomelania hupensis hupensis* snails as described [16]. In order to obtain schistosomula, cercariae were passed through a 22-gauge emulsifying needle 25 times to mechanically shear the cercarial tails from the bodies. The resulting larvae were separated from the free tails by centrifugation, washed three times with a modified Basch’s medium [17] and incubated at 37 °C under a 5 % CO_2_ atmosphere before experimentation.

### Cloning *S. japonicum* AChE

A Qiagen RNeasy kit (Qiagen, Hilden, Germany) was used to purify total RNA from adult *S. japonicum.* A one step RT-PCR (Qiagen) kit was employed to amplify specific cDNA. Based on the conserved sequences of AChE in *S. mansoni* (AF279461), S*. haematobium* (AF279462) and *S. bovis* (AF279463), and partial *S. japonicum* sequences available at http://www.genedb.org/Homepage/Sjaponicum, four pairs of primers for *Sj*AChE were designed (Table 1) to obtain the full-length cDNA sequence to PCR amplify the full-length sequence of *Sj*AChE using an overlap strategy.

**Table 1:** Primers used in PCR to obtain the full-length cDNA sequence encoding *S. japonicum* acetylcholinesterase

Primer ID	Primer pair sequence (5′-3′)		
	Forward	Reverse	Size (bp)
*Sj*AChE1	ACATGTAGATTCATTCACTATGAAAATG	ACATTGGAGTATTTGGTACCCAC	655
*Sj*AChE2	AAATTACCAGCCAGTTGTCCA	CCGCCGAAATCTTCAATGTG	404
*Sj*AChE3	TTTCTTTATATGAACACAGAAGAAGCACCAGGTAA	TTCATAACCATGCATTGTACCAGTCC	936
*Sj*AChE4	ACAGCTGTAACAAATGATTATCGTATACCAGGTC	CACGCCTAAACAATGCTGACGATT	561

### Sequence and phylogenetic analysis

Searches for homologous acetylcholinesterase protein sequences were performed using BLAST on the NCBI web site (http://blast.ncbi.nlm.nih.gov/Blast.cgi) and the WormBase ParaSite web site (http://parasite.wormbase.org/Multi/Tools/Blast). Phylogenetic analysis was performed using online resources (http://www.phylogeny.fr/simple_phylogeny.cgi) [18] by uploading the set of available AChE sequences from the different species presented. Molecular weight and isoelectric point determinations were performed using the ExPASy-Compute pI/Mw tool (http://web.expasy.org/compute_pi/). The PHYRE2 protein fold recognition server (http://www.sbg.bio.ic.ac.uk/phyre2/) was used to generate the three-dimensional (3D) model of *Sj*AChE [19] and binding site predictions were carried out using the 3DLigandSite (http://www.sbg.bio.ic.ac.uk/3dligandsite/) [20].

### Protein expression, purification and antibody generation

A C-terminal fragment of *Sj*AChE (from Q465 to V680, named *Sj*AChEC) was amplified and cloned into the pET28b vector (Novagen, Madison, USA), by using forward (5′-CGG GAT CCT CAG TTG CCG ACA CTT GAA AGT TGG A-3′ with *Bam*HI restriction site underlined) and reverse (5′-CGC TCG AGC ACG CCT AAA CAA TGC TGA CGA TTA CG-3′ with *Xho*I restriction site underlined) primers. The reconstructed vector was then transformed into *Escherichia coli* (BL21 strain) for expression induced with 1 mM IPTG (isopropyl thio-b-D-galactoside) at 37 °C for 3 h. Recombinant protein was purified from inclusion bodies by chromatography using a Ni-NTA His-tag affinity kit (Novagen) under denaturing conditions using 6 M guanidine according to the manufacturer’s instructions.

Antibodies were raised against the *Sj*AChEC fusion protein in a rabbit at the South Australian Health and Medical Research Institute (SAHMRI). Briefly, the rabbit was immunized three times each with 500 μg recombinant protein at three week intervals. Based on the fact that complete Freund’s adjuvant is the most effective adjuvant available for consistently producing high titer antibodies to diverse antigens, we used complete Freund’s adjuvant in the initial injection, but in the subsequent two used incomplete Freund’s adjuvant. The injections were delivered subcutaneously at multiple sites along the neck and spine. Blood was collected two weeks after the final boost. The titre of the antibody was determined using an enzyme-linked immunosorbent assay (ELISA). Briefly, Maxisorb immunoplates (Nalge Nune International, USA) were coated overnight at 4 °C with r*Sj*AChE protein (100 μl of 0.5 μg/ml) in coating buffer (100 μl/well). After three washes with 0.05 % (v/v) Tween in PBS (PBST), wells were blocked with 200 μl of 5 % (v/v) skim milk in PBS (SMP) and incubated for 1 h at 37 °C. The rabbit anti-SjAChE serum was serially diluted (from 1:200 to 1:102,400) in SMP and 100 μl in duplicate of each dilution were added to individual wells. After incubation at 37 °C for 1 h, the wells were washed with PBST (3X) and 100 μl (1:2,000 dilution) of horseradish peroxidise (HRP)-conjugated goat anti-rabbit IgG (Invitrogen) was added. After incubation at 37 °C for 1 h, the wells were washed with PBST (5X), 100 μl of substrate solution [2,2-azino-di-(ethyl-benzithiozolin sulfonate)] (Sigma, Castle Hill, Australia) was added and the wells were incubated at room temperature and read on a plate reader by using Microplate manager software (Bio-Rad, Mississauga, Canada). Data are presented as antibody endpoint titres, defined as the highest dilution of test serum that yielded an average O.D. two standard deviations (SDs) greater than that obtained in the absence of primary antibody.

### Western blot analysis

The rabbit anti-*Sj*AChEC serum was used in Western blotting to probe to the electrophoresed purified recombinant *Sj*AChEC protein and the native *Sj*AChE protein in a separated crude *S. japonicum* antigen extract. The crude antigen was prepared from adult worms of *S. japonicum* freshly perfused from mice percutaneously infected with 60 cercariae six weeks previously. After three washes in perfusion buffer (8.5 g NaCl and 15 g NaCitrate in 1 l of water), to minimise contamination of the schistosome protein extract with host components, an adult worm antigen preparation (SWAP) was made as described [21]. The recombinant *Sj*AChEC and SWAP samples were separated on a 15 % (w/v) SDS-PAGE gel and transferred to an Immun-Blot® low fluorescence-PVDF membrane. Overnight blocking was performed with Odyssey buffer at 4 °C. Then, the membrane was subjected to incubation with the rabbit anti-*Sj*AChE anti-serum (1:100 dilution in Odyssey buffer and 0.1 % Tween-20) for 1 h followed by incubation with IRDye-labeled 680LT goat anti-rabbit IgG antibody (Li-COR Biosciences) (1:15,000 diluted in Odyssey buffer with 0.1 % Tween-20 and 0.01 % SDS) for 1 h on a shaker in a dark chamber. After a final wash with distilled water, the membrane was allowed to dry in the dark and visualized using the Odyssey® CLx Infrared Imaging System [22].

### Immunolocalisation

#### Adult S. japonicum

Horseradish peroxidise (HRP) labelling was used for the immunolocalisation of *Sj*AChE in adult *S. japonicum*. Freshly perfused male and female worms were fixed in 100 % methanol, embedded in Tissue-Tek Optimal Cutting Temperature (OCT) compound (ProSciTech, Queensland, Australia), and 7.0 μm cryostat sections produced. The HRP labelling was performed according to standard procedures [21]. The primary antibody solution was a 1:200 dilution of the rabbit anti-*Sj*AChE serum, and normal rabbit serum was used as control. Non-specific antibody binding was inhibited by incubating the section in 10 % (v/v) normal goat serum in PBS. ImmPRESS^TM^ HRP Anti-Rabbit IgG (Peroxidase) Polymer (Vector Labs, California USA) was used as second antibody for the immunolocalisation. Slides were scanned and digitised using a ScanScope XT (Aperio, California, USA).

#### Schistosomula

Four-day old transformed larvae were cultured in Basch’s medium [3] containing rabbit anti-*Sj*AChEC serum (1:100 dilution), or pre-immune rabbit serum (1:100 dilution) as negative control, at 4 °C overnight [23]. The larvae were washed three times with Basch medium and incubated with 1:300 donkey anti-rabbit IgG Alexa Fluor 555 (2 mg/ml, Invitrogen) for 1 h at room temperature, followed by three further washes in the medium. The larvae were fixed in 4 % paraformaldehyde in PBS for 10 min at room temperature, and then visualised under fluorescence using a Zeiss 780 NLO confocal microscope (Zeiss, Germany).

### Fluorescence-based enzyme assays

The enzymatic activity of AChE in *S. japonicum* was determined using the Amplex Red Acetylcholine/Acetylcholinesterase Assay Kit (Invitrogen) according to the manufacturer’s instructions. The assay is fluorescence-based and utilises the highly fluorescent end product resorufin which is processed in black Costar 96-well plates (Sigma) and measured using the POLARstar OPTIMA (BMG Labtech, Ortenberg Germany) at an absorption of 560 nm and an emission of 590 nm. Negative and positive control samples are provided in the assay kit and BW284c51 [1,5-bis(4allyldimethylammoniumphenyl)pentan-3-one dibromide] (Sigma), a specific inhibitor of AChE, was also used in the enzyme assay.

Different protein extracts of *S. japonicum* used in the enzymatic assays:

(1) Tegument protein and residual carcass protein extracted from adult *S. japonicum* freshly perfused from mice. The tegument was removed from paired adult worms by the freeze/thaw/vortex method [24]. Briefly, freshly perfused paired adult *S. japonicum* (50 pairs) were frozen in liquid nitrogen, thawed on ice and 400 μl of ice-cold TBS (10 mM Tris-HCl, 0.84 % NaCl, pH 7.4) was added to each tube. The supernatant was removed after 1 min and then 400 μl of Tris-HCl, pH 7.4 was added and the tube left to incubate on ice for 5 min. Tubes were vortexed 8 times for 1 s to ensure tegument release. The tegument-rich supernatant was transferred to another tube where it was centrifuged for 30 min at 12,000 *g*, 4 °C, the supernatant was discarded and the tegument-rich pellet re-suspended in 60 μl 10 mM Tris-HCl, pH 8.0. The remaining carcasses were homogenised using the protocol for making SWAP essentially as described in [21], and above. The protein concentrations of the enriched tegument fraction and the residual carcass preparation were measured using the Bio-Rad protein assay dye reagent (Bio-Rad, California, USA). These protein extracts (0.005 mg/ml) were pre-incubated at room temperature for 30 min with BW284c51 at concentrations of 0, 10, 100 and 1000 μM and then used in the AChE activity assays.

(ii) Freshly perfused adult *S. japonicum* were cultured in RPMI medium containing 10 % (v/v) heat-inactivated fetal calf serum overnight. The worms were then divided into three groups: single males, single females and paired male and female worms (40 single worms or 20 pairs/group). Each group was then treated with or without 200 μM BW284c51 for 24 h, after which time the worms were rinsed 3 times in RPMI medium, collected and used for tegumental protein and carcass protein extraction as described above. AChE activities of all the protein samples (0.005 mg/ml) from the various worm samples were measured using the Amplex Red Acetylcholine/Acetylcholinesterase Assay Kit.

Worm collections, protein extractions and AChE activity measurements in (i) and (ii) were performed three times. T-test was employed to make the comparison between samples by using GraphPad software (version 7.0).

## Results

### Full-length sequence of *Sj*AChE

**Fig. 1:**
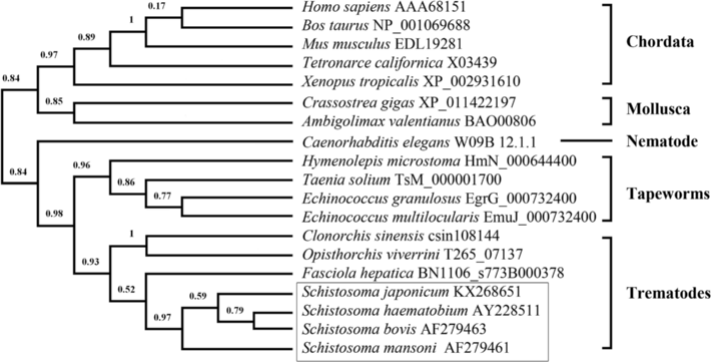
Phylogenetic analysis of *S. japonicum* acetylcholinesterase and functionally characterised acetylcholinesterases from other taxa. Species (Sequence Accession Number) used for the analysis include: *Mus musculus* (EDL19281), *Bos taurus* (NP_001069688), *Homo sapiens* (AAA68151), *Tetronarce californica* (X03439), *Xenopus tropicalis* (XP_002931610), *Ambigolimax valentianus* (BAO00806), *Crassostrea gigas* (XP_011422197), *Caenorhabditis elegans* (W09B12.1.1*), *Hymenolepis microstoma* (HmN_000644400*), *Taenia solium* (TsM_000001700*), *Echinococcus granulosus* (EgrG_000732400*), *Echinococcus multilocularis* (EmuJ_000732400*), *Clonorchis sinensis* (csin108144*), *Opisthorchis viverrini* (T265_07137*), *Fasciola hepatica* (BN1106_s773B000378), *Schistosoma. haematobium* (AF279462), *S. bovis* (AF279463), *S. mansoni* (AF279461). Note: *: Gene ID from WormBase ParaSite web site (http://parasite.wormbase.org/Multi/Tools/Blast)

The complete *Sj*AChE cDNA sequence was obtained, comprising an open reading frame (ORF) of 2,040 bp (submitted to GenBank under accession number KX268651) encoding 680 amino acids. *Sj*AChE shares 88 % amino acid sequence identity with the AChEs from *S. mansoni,* S*. haematobium* and *S. bovis,* and 55 % identity with the AChEs in *Echinococcus granulosus* [25] and *E. multilocularis* [26]. In contrast, *Sj*AChE shares only 25 % amino acid identity to human AChE and 26 % identity to the AChE from *Torpedo californica* (Pacific electric ray). Phylogenetic analysis was performed using AChE protein sequences from a variety of species to produce a cladogram to infer evolutionary relationships between taxa (Fig. 1). Of the schistosome sequences, the AChE coding region for *S. japonicum* is most similar to that of *S. haematobium*. The *Schistosoma* spp. sequences are separated considerably from those of *Echinococcus* species and, as expected, have more sequence similarity with the AChEs of other trematode species including *Clonorchis sinensis*, *Opisthorchis viverrini* and *Fasciola hepatica*. Crucially, all residues within the AChE protein sequence that are currently known to be important for substrate binding and catalytic activity, i.e. those comprising the peripheral anionic site [27], the catalytic triad substrate inhibition of acetylcholinesterase residues involved in signal transduction from the surface to the catalytic center [28], and those lining the catalytic gorge [29], are conserved across all *Schistosoma* species.

**Fig. 2:**
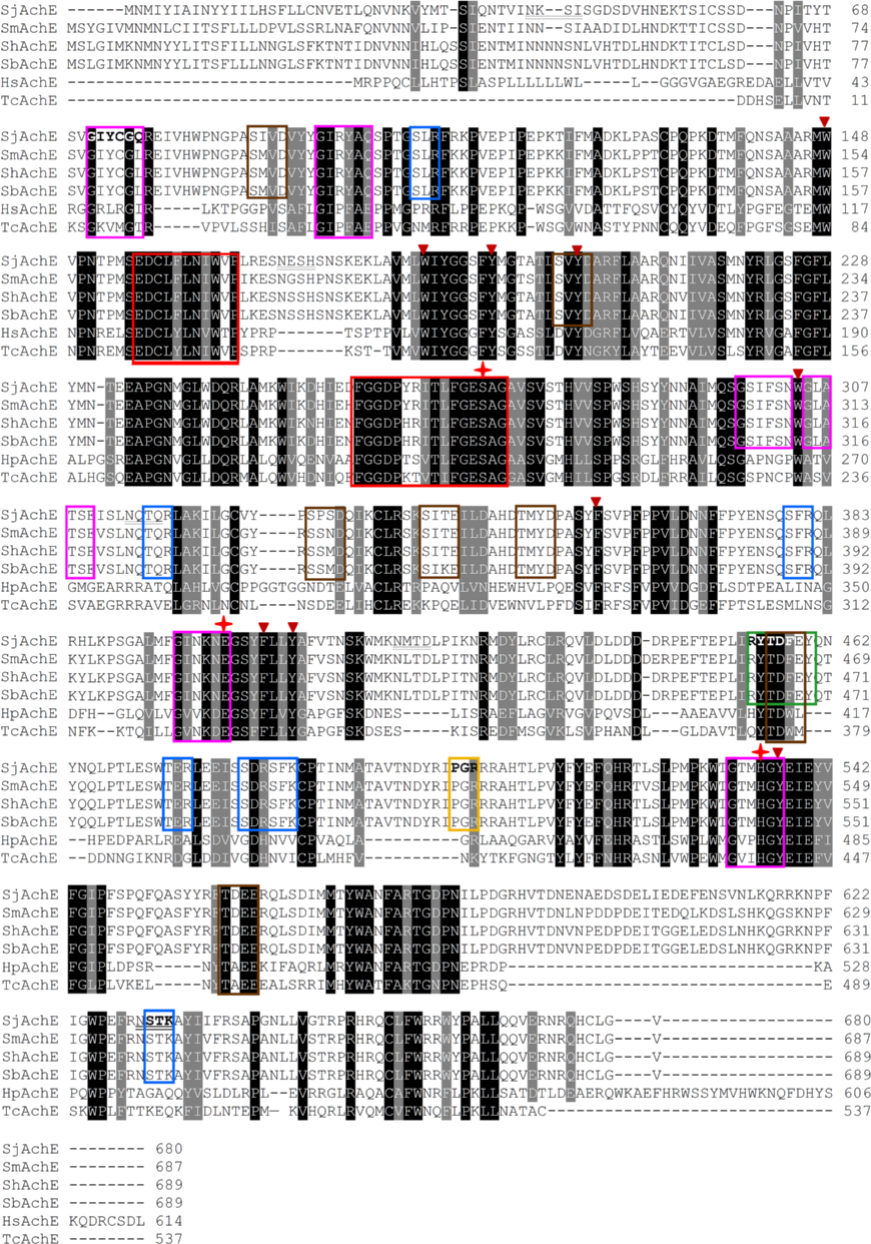
Alignment of acetylcholinesterases from *S. mansoni*, S. *haematobium*, *S. bovis, Homo sapiens* and *T. californica*. Red boxes indicate the two conserved subdomains including carboxylesterase type-B signature 2 (E156-P166) and carboxylesterase type-B serine active site (F258-G273). Several motifs are found in *Sj*AChE: N-glycosylation sites underlined (N42-I45, N171-H174, N314-Q317, N418-D421, N630-K633); N-myristoylation sites boxed in purple (G71-Q76, G95-Q100, G298-N303, G305-E310, G395-E400, G532-Y537); Casein kinase II phosphorylation site boxed in brown (S88-D91, S200-D203, S329-D332, S341-E344, T351-D354, T456-E459, S471-E474, T559-E562, T592-E595); Protein kinase C phosphorylation sites boxed in blue (S105-R107, T316-R318, S379-R381, T473-R475, S481-K486, S631-K633) which are specific for schistosome; Tyrosine kinase phosphorylation site boxed in green (R454-Y460); amidation site (P503-R506). The conserved catalytic active catalytic triad site is observed *S. japonicum* (S280-H54-E327, in red stars), while the 9 residues (W148, W186, W193, Y202, W304, F371, F404, Y407, Y537, in dark red triangles) in the rings of 14 aromatic amino acid residues of *T. californica* AChE are conserved in the appropriate locations in *S. japonicum* AChE. The coloured boxes which covered only sequences from four species of schistosomes indicated the specific motifs for schistosome. Note: AChE from *S. mansoni* (SmAChE), S. *haematobium* (ShAChE), *S. bovis* (SbAChE), *Homo sapiens* (HsAChE) and *T. californica* (TcAChE)

Using an *in silico* motif and domain search tool (http://prosite.expasy.org/), we identified two conserved sub-domains in the *Sj*AChE protein sequence - a carboxylesterase type-B signature 2 (E156-P166) region, and a carboxylesterase type-B serine active site (F258-G273), both of which are shown boxed in red in Fig. 2. Several other motifs were also found in *Sj*AChE (Fig. 2); these included:

1. N-glycosylation sites underlined (N42-I45, N171-H174, N314-Q317, N418-D421, N630-K633);
2. N-myristoylation sites boxed in purple (G71-Q76, G95-Q100, G298-N303, G305-E310, G395-E400, G532-Y537);
3. A casein kinase II phosphorylation site boxed in brown (S88-D91, S200-D203, S329-D332, S341-E344, T351-D354, T456-E459, S471-E474, T559-E562, T592-E595);
4. Protein kinase C phosphorylation sites boxed in blue (S105-R107, T316-R318, S379-R381, T473-R475, S481-K486, S631-K633) which are specific for schistosomes;
5. A tyrosine kinase phosphorylation site boxed in green (R454-Y460);
6. An amidation site boxed in yellow (P503-R506).

After comparisons with other species and the schistosome sequences published by Bentley et al [30], we demonstrated that the catalytic and peripheral active site residues in *S. japonicum*, *S. mansoni*, *S. haematobium* and *S. bovis* are all conserved, especially when taking into consideration the accepted standard primary AChE (1EA5_A) sequence from the ray *Torpedo californica.* It has been shown that the active site of *T. californica* AChE consists of a catalytic triad (S200-H440-E327, in red stars, Fig. 2) which lies close to the bottom of a deep and narrow tertiary structure gorge, which is lined with the rings of 14 aromatic amino acid residues [31]. The conserved catalytic triad is present in *S. japonicum* (S280-H54-E327), while the nine residues (W148, W186, W193, Y202, W304, F371, F404, Y407, Y537, in dark red triangles, Fig. 2) in the rings of the 14 aromatic amino acid residues of *T. californica* AChE, are conserved in the appropriate locations in *Sj*AChE.

**Fig. 3:**
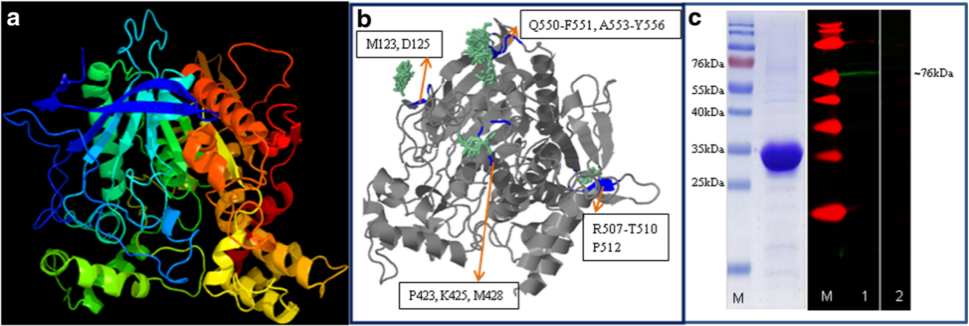
**a** Three dimensional model of *S. japonicum* acetylcholinesterase determined using PHYRE2. Image coloured by rainbow from N to C terminus, Model dimensions (Å) X: 61.705 Y: 62.361 Z: 71.856 are the same as that of ShAChE. **b** The predicted binding sites of *Sj*AChE with *N*-Acetylglucosamine (NAG). The four predicted *N*-Acetylglucosamine binding sites (in blue) are located at (i) M123, D125; (ii) P423, K245, M428; (iii) R507-T510, P512; and (iv) Q550-F551, A553-Y556 in *Sj*AChE. The NAG residues are shown in green. **c** Western blot analysis using anti- *Sj*AChEC to detect the total extracts from adult *S. japonicum. Left panel*: SDS-PAGE gel of purified recombinant protein *Sj*AChEC (Molecular size: 30 Kda); *Right panel*: western blot analysis of total extract from adult *S. japonicum* worms. The protein extract was probed with rabbit anti-*Sj*AChEC antibody (Lane 1) by recognising a band of approximately 76 kDa which match the calculated molecular size for native *Sj*AChE Pre-immune sera (Lane 2) was used as control. Lane M, PageRuler^TM^ pre-stained protein ladder

The tertiary protein structure for *Sj*AChE was predicted using Phyre2 (Fig. 3a). Model dimensions for *Sj*AChE (Å) (X:61.705 Y:62.361 Z:71.856) are the same as those of *S. haematobium* AChE. Of note, we found four predicted *N*-Acetylglucosamine (NAG) binding sites located at (i) M123, D125; (ii) P423, K245, M428; (iii) R507-T510, P512; and (iv) Q550-F551, A553-Y556 in *Sj*AChE (Fig. 3b). *N*-Acetylglucosamine, a monosaccharide derivative of glucose, is directly incorporated into glycosaminoglycans and glycoproteins, acting as a substrate for tissue repair mechanisms [32]. The predicted four NAG binding sites in *Sj*AChE are in line with previous findings which revealed the presence of NAG in all forms of cholinesterases investigated [20], providing evidence for N-linked glycosylation in *Sj*AChE. The predicted protein structure for *Sj*AChE also suggests that it may be fucosylated on the innermost N-acetylglucosamine residue of the core [33].

### Western-blot analysis

SDS-PAGE showed the purified r*Sj*AChEC migrated as a single band with the predicted size of 30 kDa (Fig. 3c). The specificity of the rabbit anti-*Sj*AChEC antibody was confirmed as it bound a band of approximately 76 kDa in adult *S. japonicum* SWAP, thereby matching well with the calculated molecular size for *Sj*AChE (Fig. 3c). Control serum from the pre-immunized control rabbit did not bind any protein component in *S. japonicum* SWAP.

### Distribution of *Sj*AChE in adults and schistosomula

**Fig. 4:**
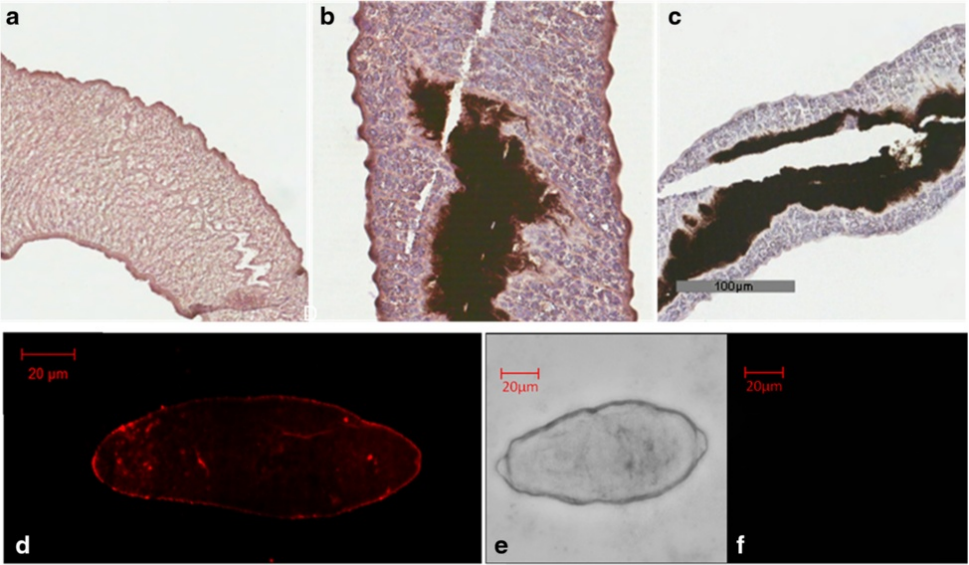
Immunolocalisation of *Sj*AChE in adult *S. japonicum* and four-day mechanically transformed schistosomula. Adult male **a** and female **b** worm sections were labelled with rabbit anti-*Sj*AChE antibody coupled with anti-rabbit HRP and scanned using an Aperio scanner. **c** Negative control sections of female worm were incubated with rabbit pre-immune serum. The female gut (**b**, **c**) appears non-specifically opaque due to the presence of red blood cell products, with the histochemical negative control demonstrating the dark region is due to gut contents. **d** Immunofluorescence of *Sj*AChE in four-day old schistosomula probed with rabbit anti-*Sj*AChE antibody. **e** Brightfield and corresponding fluorescence images **f** of schistosomula negative controls using pre-immune rabbit serum. Donkey anti-rabbit IgG 555 was used as secondary antibody and positive immunofluorescence is shown in red. As a negative control, **f** shows there is no signal produced when incubating parasites with rabbit pre-immune serum in the adult worm tissue. *Scale-bars*: a-c, 100 μm; d-f, 20 μm

Indirect immunohistochemistry, incorporating HRP labelling, indicated that *Sj*AChE immunoreactivity occurred in the tegument, the underlying musculature but also throughout the parenchyma and tissues of both males (Fig. 4a) and females (Fig. 4b)*.* To better understand how the anti-*Sj*AChE serum interacted with schistosomula, we used immunofluorescence to show *Sj*AChE is also localized on the tegumental surface of live 4-day-old schistosomula (Fig. 4d) and the parenchyma; the latter observation may be indicative of damage to the schistosomula during labelling process. By using two different immunolocalisation methods involving HRP labelling and immunofluorescence, we showed a similar distribution of *Sj*AChE in early (schistosomula) and late (adult males and females) developmental stages in the mammalian host.

### Inhibition of *Sj*AChE activity

**Fig. 5:**
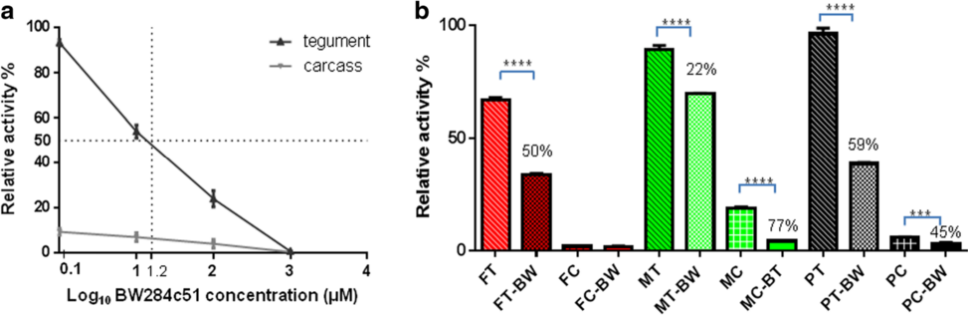
Inhibition of AChE activity in adult *Schistosoma japonicum* protein preparations or intact worms. **a** *Sj*AChE activity in the tegument extract was significantly higher (about 10-fold; *P <* 0.0001) than in the carcasses of adult worms. The IC50 (50 % inhibition) of BW284c51 on *Sj*AChE in the tegumental protein extract of adult worms is indicated as dotted lines at a concentration of 16 μM. **b** The sensitivity of *Sj*AChE in the tegument and carcasses, isolated from cultured male and female worms in the presence of 200 μM BW284c51, were different. At the same concentration of tegument protein, paired worms had higher *Sj*AChE activity than single-sex worms with male worms (*P* = 0.0175) having a higher *Sj*AChE activity than females (*P <* 0.0001). After treatment with BW284c51, *Sj*AChE enzyme activity in tegument protein extracts of paired worms, male worms and female worms decreased by 59 %, 22 % and 50 %, respectively (*P <* 0.0001). Compared with the tegumental protein extract, there was much less *Sj*AChE activity in the carcass protein extract, with a relatively higher activity in males. Error bars represent the standard error of the mean (SEM). These experiments were performed three times (*n* = 3). Relative activity (%) = 100 × (sample fluorescence – negative fluorescence)/(positive–negative fluorescence). *P ≤ 0.05, **P ≤ 0.001, ***P ≤ 0.0001

*Sj*AChE sensitivity to chemical inhibition, in extracts of adult worms, was assessed by the pre-incubation of tegument or carcass proteins with BW284c51 at a concentration range of 0–1,000 μM. *Sj*AChE, present both in the worm tegument or carcass extract, was sensitive to BW284c51, and its activity exhibited a linear response to concentration changes up to 1000 μM of BW284c51 (Fig. 5a). *Sj*AChE activity in the tegument extract was significantly higher (about 10-fold; t-test, *t* = 1.881, *df* = 6, *P <* 0.0001) than in the carcasses of adult worms, suggesting the majority of the enzyme is located on the tegument of paired adult *S. japonicum*. The IC50 (50 % inhibition) of BW284c51 on *Sj*AChE in the tegumental protein extract of adult worms occurred at a concentration of 16 μM which indicates a substantially higher sensitivity than that reported for the AChEs from *S. mansoni*, *S. bovis* and *S. haematobium* [2]. The sensitivity of *Sj*AChE in the tegument and carcasses isolated from cultured adult worms in the presence of 200 μM BW284c51 (IC80, 80 % inhibitory concentration) are shown in Fig. 5b. With the same concentration of tegument protein, paired worms had a higher *Sj*AChE activity than single-sex worms (t-test, *t* = 3.903, *df* = 4, *P* = 0.0175) with male worms having a higher *Sj*AChE activity than females (t-test, *t* = 18.66, *df* = 4, *P <* 0.0001). After being treated with 200 μM BW284c51, the *Sj*AChE enzyme activity in tegument protein extracts of paired worms, males and female worms decreased by 59 %, 22 % and 50 %, respectively (t-test, *t* = 40.52,; 17.28; and 39.56, respectively, *df* = 4, *P <* 0.0001). Compared with the tegumental protein extract, there was much less *Sj*AChE activity in the carcass protein extract, with a relatively higher activity in males compared with that in pairs and females (t-test, *t* = 29.41 and 39.07, respectively, *df* = 4, *P <* 0.0001), with the latter having the lowest level of *Sj*AChE activity. *Sj*AChE activity in the carcass protein extracts of males and paired worms was inhibited by 77 % (t-test, *t* = 32.69, *df* = 4, *P <* 0.0001) and 45 % (t-test, *t* = 15.07, *df* = 4, *P <* 0.0001), respectively in the presence of 200 μM BW284c51.

## Discussion

Previous studies on AChE in schistosomes have focused mainly on *S. mansoni*, *S. haematobium* and *S. bovis* and, prior to this study, very limited information was available for the enzyme in *S. japonicum*. Here, we report the cloning and expression of the complete cDNA encoding *S. japonicum* AChE (*Sj*AChE)*.* To better understand its functions, we performed sequence and phylogenetic analysis on *Sj*AChE and predicted its tertiary molecular structure. As might be expected, the protein has high sequence identity (88 %) with the AChEs in *S. mansoni*, *S. haematobium* and *S. bovis*. The key residues that are important for the formation of the three disulphide bonds and two salt bridges characteristic of AChE [29], in substrate binding and for catalytic activity are conserved across the four species. These residues comprise important structural features including the peripheral anionic site [27], the catalytic triad [28] and residues that line the catalytic gorge [29]. One particularly noteworthy feature of the AChE protein sequence in schistosomes is two “missing” residues that form part of the peripheral active site. Within the AChE of *Torpedo californica*, residue F330 has a neighbouring F residue in the same secondary structure that is not indicated as playing a role in the catalysis of acetylcholine. However, whereas in schistosomes, the equivalent of F330 is not present (Fig. 2), the neighbouring F residue is. It may be possible that this neighbouring F residue has taken over the catalytic role, or that this role has been lost altogether in schistosomes. Similarly, an equivalent residue could not be found at the position expected for W279 (Fig. 2), another peripheral active site residue. Considering these residues are only part of the peripheral active site, they may not be essential for the function of AChE in schistosomes and have been lost over time through mutational events.

As with the other schistosomes, immunolocalisation showed that *Sj*AChE is located on the tegumental surface and parenchyma of adult worms and 4-day-old schistosomula [2]. Previous work showed the existence of two principal molecular forms (external and internal) of *S. mansoni* AChE, with approximately half of the AChE activity being found on the tegumental membrane via a covalently attached glycosylphosphatidylinositol (GPI) anchor and which may function in signal transduction, with the remainder mainly associated with muscle tissue and involved in cholinergic processes [34]. These two forms of AChE were also shown to differ in their heparin-binding properties (only the internal form interacted with heparin) and in immunological specificity (being located on the surface the GPI-anchored form may be susceptible as an immunological target) [35]. Further investigation is required to determine whether there are also different molecular forms of *Sj*AChE and if so whether they have discrete functional roles in *S. japonicum*.

To quantify the relative activity of *Sj*AChE present within the tegument and in the musculature of adult *S. japonicum*, we separated the tegumental protein from the parasite carcass, and performed enzyme activity assays. We found that most of the *Sj*AChE activity was concentrated in the tegument, having 10-fold the activity of the carcass (Fig. 5a), suggesting that *Sj*AChE has potential as a drug or immunological target*.* We also showed that *Sj*AChE activity was highly enriched in the male tegument and this observation is understandable as male parasites, being larger in size, and having an increased tegumental volume [36]. One established function of tegumental AChE in schistosomes is in the regulation of glucose uptake across the tegument in response to ACh present in the mammalian host bloodstream [7]. Given that male schistosomes play a more important role in host glucose uptake [37], it is reasonable to consider that AChE activity would also be higher in male *S. japonicum*, as we have shown. The distribution of AChE in *S. japonicum* we established correlates with that reported in the other schistosome species [13, 38].

It has been shown that AChE activity and its sensitivity to the inhibitor BW284c51 is dependent on the relative amount of AChE expressed on the surface of adult schistosomes [2], since the inhibitor does not readily penetrate membranes of the adult worms [2]. We showed a protein extract of the tegument of adult *S. japonicum* had an IC50 with BW284c51 of 16 μM, which is much lower than the reported IC50 for other schistosome species (0.1–5.0 mM) [2]. Those results may reflect a relatively larger amount of AChE activity presented on the surface of adult *S. japonicum* compared to the other schistosome species, indicating the AChE inhibitor may be more effective against *S. japonicum*. We also found that live adults of *S. japonicum* incubated with Bw284c51 (200 μM) displayed reduced AChE activity in tegumental protein by 50 % in females, but only 22 % in males, suggesting that AChE present on the surface of females is more sensitive to the inhibitor than that on males. Previous work has shown that AChE is associated with the AChR on cell surfaces [39] and in schistosomes the expression of AChR is increased in sexually paired worms when female parasites mature into the egg producing stage [13]. The increased level of AChR expression may require increased AChE activity on the surface of female worms to maintain cholinesterase receptor fidelity. A similar situation occurred in paired worms, where a 59 % decrease in *Sj*AChE activity was observed when paired incubated worms were treated with 200 μM of Bw284c51.

The relatively high level of *Sj*AChE activity distributed within the carcass protein of males, when compared with female and paired worms, may be indicative of its involvement in muscle function [34], since male worms have more muscle tissue. The *Sj*AChE activity in male carcasses was decreased by 77 % after incubation of live male parasites with BW284c51 for 24 h, suggesting that the inhibitor can penetrate the tegument of male *S. japonicum*, which is a contradiction to previous reports stating the inhibitor cannot cross membranes [2].

It has been reported that AChE expression is induced during apoptosis and is regulated by the mobilization of intracellular Ca2+ in various mammalian cell types [40]. Promoting apoptosis appeared to be a feature of the mode of action of two already established anti-schistosomal drugs, the artemisinins [41] and praziquantel [42], and drug targeting schistosome AChE may also be effective by inducing apoptosis. Further, it has been demonstrated that purified polyclonal antibodies raised against *S. mansoni* AChE were cytotoxic and caused almost total complement-dependent killing of parasites in vitro [9, 35], while not cross-reacting with human AChE. This observation and the results presented here strengthen the view that immunological targeting of schistosome AChEs may be a highly suitable avenue for future vaccine development and the prevention of schistosomiasis.

## Conclusions

In this paper, we have described the phylogenetic and molecular/structural characterisation of the AChE protein from *S. japonicum*. These findings improved the understanding of the biological function of AChE in schistosomes. The relative abundance of AChE activity (90 %) present on the surface of adult *S. japonicum* when compared with that reported in other schistosomes, suggests *Sj*AChE may be a more effective drug or immunological target against thus species*.* Furthermore, we show that the AChE activity in tegumental extracts of adult *S. japonicum* can be significantly inhibited by the classical inhibitor of AChE (BW285c51) after incubation with adult worms. The results we present support the potential of AChE as a future drug target against *S. japonicum* and also strengthens the view that immunological targeting of schistosome AChEs may be a highly suitable avenue for future vaccine development and the prevention of schistosomiasis.

## Abbreviations

ACh, acetylcholine; AChE, acetylcholinesterase; ELISA, enzyme-linked immunosorbent assay; GPI, glycosylphosphatidylinositol; HRP, horseradish peroxidise; nAChR, nicotinic acetylcholine receptor; NAG, *N*-Acetylglucosamine; PSMD4, proteasome non-ATPase regulatory subunit 4; *Sj*AChE, *Schistosoma japonicum* acetylcholinesterase; SWAP, soluble adult worm antigen preparation

## References

[1] Beaumier CM, Gillespie PM, Hotez PJ, Bottazzi ME (2013). New vaccines for neglected parasitic diseases and dengue. Transl Res.

[2] Camacho M, Tarrab-Hazdai R, Espinoza B, Arnon R, Agnew A (1994). The amount of acetylcholinesterase on the parasite surface reflects the differential sensitivity of schistosome species to metrifonate. Parasitology.

[3] Salafsky B, Fusco AC, Whitley K, Nowicki D, Ellenberger B (1988). *Schistosoma mansoni*: analysis of cercarial transformation methods. Exp Parasitol.

[4] Wilson RA (2012). Proteomics at the schistosome-mammalian host interface: any prospects for diagnostics or vaccines?. Parasitology.

[5] Levi-Schaffer F, Tarrab-Hazdai R, Schryer MD, Arnon R, Smolarsky M (1984). Isolation and partial characterization of the tegumental outer membrane of schistosomula of *Schistosoma mansoni*. Mol Biochem Parasitol.

[6] Ribeiro-dos-Santos G, Verjovski-Almeida S, Leite LC (2006). Schistosomiasis - a century searching for chemotherapeutic drugs. Parasitol Res.

[7] Camacho M, Agnew A (1995). *Schistosoma*: rate of glucose import is altered by acetylcholine interaction with tegumental acetylcholine receptors and acetylcholinesterase. Exp Parasitol.

[8] Harder A (2002). Chemotherapeutic approaches to schistosomes: current knowledge and outlook. Parasitol Res.

[9] Espinoza B, Tarrab-Hazdai R, Himmeloch S, Arnon R (1991). Acetylcholinesterase from *Schistosoma mansoni*: immunological characterization. Immunol Lett.

[10] Bentley GN, Jones AK, Agnew A (2005). Expression and comparative functional characterisation of recombinant acetylcholinesterase from three species of *Schistosoma*. Mol Biochem Parasitol.

[11] Jones AK, Bentley GN, Oliveros Parra WG, Agnew A (2002). Molecular characterization of an acetylcholinesterase implicated in the regulation of glucose scavenging by the parasite *Schistosoma*. FASEB J.

[12] Pearson MS, Becker L, Driguez P, Young ND, Gaze S, Mendes T, Li XH, Doolan DL, Midzi N, Mduluza T (2015). Of monkeys and men: immunomic profiling of sera from humans and non-human primates resistant to schistosomiasis reveals novel potential vaccine candidates. Front Immunol.

[13] Camacho M, Alsford S, Jones A, Agnew A (1995). Nicotinic acetylcholine receptors on the surface of the blood fluke *Schistosoma*. Mol Biochem Parasitol.

[14] Bentley GN, Jones AK, Oliveros Parra WG, Agnew A (2004). ShAR1alpha and ShAR1beta: novel putative nicotinic acetylcholine receptor subunits from the platyhelminth blood fluke *Schistosoma*. Gene.

[15] Bentley GN, Jones AK, Agnew A (2007). ShAR2beta, a divergent nicotinic acetylcholine receptor subunit from the blood fluke *Schistosoma*. Parasitology.

[16] Jones MK, McManus DP, Sivadorai P, Glanfield A, Moertel L, Belli SI, Gobert GN (2007). Tracking the fate of iron in early development of human blood flukes. Int J Biochem Cell Biol.

[17] Gobert GN, Tran MH, Moertel L, Mulvenna J, Jones MK, McManus DP, Loukas A (2010). Transcriptional changes in *Schistosoma mansoni* during early schistosomula development and in the presence of erythrocytes. PLoS Negl Trop Dis.

[18] Dereeper A, Guignon V, Blanc G, Audic S, Buffet S, Chevenet F, Dufayard JF, Guindon S, Lefort V, Lescot M (2008). Phylogeny.fr: robust phylogenetic analysis for the non-specialist. Nucleic Acids Res.

[19] Kelley LA, Sternberg MJ (2009). Protein structure prediction on the web: a case study using the Phyre server. Nat Protoc.

[20] Wass MN, Kelley LA, Sternberg MJ (2010). 3DLigandSite: predicting ligand-binding sites using similar structures. Nucleic Acids Res.

[21] You H, Zhang W, Jones MK, Gobert GN, Mulvenna J, Rees G, Spanevello M, Blair D, Duke M, Brehm K (2010). Cloning and characterisation of *Schistosoma japonicum* insulin receptors. PLoS One.

[22] Ranasinghe SL, Fischer K, Gobert GN, McManus DP (2015). Functional expression of a novel Kunitz type protease inhibitor from the human blood fluke *Schistosoma mansoni*. Parasit Vectors.

[23] McWilliam HE, Driguez P, Piedrafita D, Maupin KA, Haab BB, McManus DP, Meeusen EN (2013). The developing schistosome worms elicit distinct immune responses in different tissue regions. Immunol Cell Biol.

[24] Jia X, Schulte L, Loukas A, Pickering D, Pearson M, Mobli M, Jones A, Rosengren KJ, Daly NL, Gobert GN (2014). Solution structure, membrane interactions, and protein binding partners of the tetraspanin Sm-TSP-2, a vaccine antigen from the human blood fluke *Schistosoma mansoni*. J Biol Chem.

[25] Zheng H, Zhang W, Zhang L, Zhang Z, Li J, Lu G, Zhu Y, Wang Y, Huang Y, Liu J (2013). The genome of the hydatid tapeworm *Echinococcus granulosus*. Nat Genet.

[26] Tsai IJ, Zarowiecki M, Holroyd N, Garciarrubio A, Sanchez-Flores A, Brooks KL, Tracey A, Bobes RJ, Fragoso G, Sciutto E (2013). The genomes of four tapeworm species reveal adaptations to parasitism. Nature.

[27] Barak D, Ordentlich A, Bromberg A, Kronman C, Marcus D, Lazar A, Ariel N, Velan B, Shafferman A (1995). Allosteric modulation of acetylcholinesterase activity by peripheral ligands involves a conformational transition of the anionic subsite. Biochemistry.

[28] Shafferman A, Velan B, Ordentlich A, Kronman C, Grosfeld H, Leitner M, Flashner Y, Cohen S, Barak D, Ariel N (1992). Substrate inhibition of acetylcholinesterase: residues affecting signal transduction from the surface to the catalytic center. EMBO J.

[29] Sussman JL, Harel M, Frolow F, Oefner C, Goldman A, Toker L, Silman I (1991). Atomic structure of acetylcholinesterase from *Torpedo californica*: a prototypic acetylcholine-binding protein. Science.

[30] Bentley GN, Jones AK, Agnew A (2003). Mapping and sequencing of acetylcholinesterase genes from the platyhelminth blood fluke *Schistosoma*. Gene.

[31] Sussman JL, Harel M, Silman I (1993). Three-dimensional structure of acetylcholinesterase and of its complexes with anticholinesterase drugs. Chem Biol Interact.

[32] Salvatore S, Heuschkel R, Tomlin S, Davies SE, Edwards S, Walker-Smith JA, French I, Murch SH (2000). A pilot study of N-acetyl glucosamine, a nutritional substrate for glycosaminoglycan synthesis, in paediatric chronic inflammatory bowel disease. Aliment Pharmacol Ther.

[33] You H, McManus DP, Hu W, Smout MJ, Brindley PJ, Gobert GN (2013). Transcriptional responses of *in vivo* praziquantel exposure in schistosomes identifies a functional role for calcium signalling pathway member CamKII. PLoS Pathog.

[34] Tarrab-Hazdai R, Levi-Schaffer F, Gonzales G, Arnon R (1984). Acetylcholinesterase of *Schistosoma mansoni.* Molecular forms of the solubilized enzyme. Biochim Biophys Acta.

[35] Arnon R, Silman I, Tarrab-Hazdai R (1999). Acetylcholinesterase of *Schistosoma mansoni -* functional correlates. Contributed in honor of Professor Hans Neurath’s 90th birthday. Protein Sci.

[36] Gobert GN, Stenzel DJ, McManus DP, Jones MK (2003). The ultrastructural architecture of the adult *Schistosoma japonicum* tegument. Int J Parasitol.

[37] Cornford EM, Fitzpatrick AM (1985). The mechanism and rate of glucose transfer from male to female schistosomes. Mol Biochem Parasitol.

[38] Camacho M, Agnew A (1995). Glucose uptake rates by *Schistosoma mansoni*, *S. haematobium*, and *S. bovis* adults using a flow *in vitro* culture system. J Parasitol.

[39] Massoulie J, Pezzementi L, Bon S, Krejci E, Vallette FM (1993). Molecular and cellular biology of cholinesterases. Prog Neurobiol.

[40] Zhu H, Gao W, Jiang H, Jin QH, Shi YF, Tsim KW, Zhang XJ (2007). Regulation of acetylcholinesterase expression by calcium signaling during calcium ionophore A23187- and thapsigargin-induced apoptosis. Int J Biochem Cell Bio.

[41] Ho WE, Peh HY, Chan TK, Wong WS (2014). Artemisinins: pharmacological actions beyond anti-malarial. Pharmacol Ther.

[42] Hong Y, Donald PM, Geoffrey NG (2015). Current and prospective chemotherapy options for schistosomiasis. Expert Opin Orphan D.

